# The Course of Mechanical Stress: Types, Perception, and Plant Response

**DOI:** 10.3390/biology12020217

**Published:** 2023-01-30

**Authors:** Mohamed Kouhen, Anastazija Dimitrova, Gabriella Stefania Scippa, Dalila Trupiano

**Affiliations:** Department of Biosciences and Territory, University of Molise, 86090 Pesche, Italy

**Keywords:** calcium signaling, gravitropism, mechanosensitive channels, reaction wood, root bending, ROS signaling, slope, thigmomorphogenesis, wounding response, woody plant

## Abstract

**Simple Summary:**

Mechanical stress is a substantial natural environmental constraint for plants that is induced by dry compacted soils, intense rain and windstorms, changes in gravity, and obstacles. It is crucial to completely comprehend the precise mechanisms of plant response and adaptation to mechanical stresses as it has been demonstrated that their performance and growth rates are strongly impacted by these conditions. Over the past few decades, research in different fields (botany, biomechanics, genetics, biochemistry, imaging, etc.) has offered fragmentary insights into the mechanisms used by plants to counteract mechanical pressures. In an attempt to illustrate the complete picture, this review synthesizes current mechanical stress knowledge and research gaps on both above- and below-ground organs of annual and perennial plants, underlying similarity/differences and providing future recommendations.

**Abstract:**

Mechanical stimuli, together with the corresponding plant perception mechanisms and the finely tuned thigmomorphogenetic response, has been of scientific and practical interest since the mid-17th century. As an emerging field, there are many challenges in the research of mechanical stress. Indeed, studies on different plant species (annual/perennial) and plant organs (stem/root) using different approaches (field, wet lab, and in silico/computational) have delivered insufficient findings that frequently impede the practical application of the acquired knowledge. Accordingly, the current work distils existing mechanical stress knowledge by bringing in side-by-side the research conducted on both stem and roots. First, the various types of mechanical stress encountered by plants are defined. Second, plant perception mechanisms are outlined. Finally, the different strategies employed by the plant stem and roots to counteract the perceived mechanical stresses are summarized, depicting the corresponding morphological, phytohormonal, and molecular characteristics. The comprehensive literature on both perennial (woody) and annual plants was reviewed, considering the potential benefits and drawbacks of the two plant types, which allowed us to highlight current gaps in knowledge as areas of interest for future research.

## 1. Introduction

Plants are exposed to an ever-changing environment and is accentuated by climate deregulation, which affects plant growth, stability, and productivity. The plant’s response mechanism to altered environments consists of intricate perception mechanisms and precisely orchestrated adaptive signaling cascades, and it is this machinery that determines the plant’s capacity for adaptation and survival [1]. However, while downstream cellular signaling cascades and physiological responses have been well-explored for some types of stress, the understanding of how plants perceive signals is still debatable or problematic. In the case of mechanical stress (MS), the mechanosensory receptors, along with the plant’s rapid sensitization, are the essential evolutionary tools that plants have developed to survive and adapt [2,3,4,5]. The MS response includes a set of morphological, physiological, and biochemical adaptations. Even though these changes can take time to become apparent, they can dramatically alter the plant physiology [6]. Despite several thorough reviews that investigate MS from various aspects (e.g., [7,8,9]), the knowledge regarding the course of action of MS is dispersed. Thus, understanding the MS regulatory mechanisms could bring practical applications closer to reality by identifying the molecular targets for engineering stress-resilient species [10,11,12] as well as the classic targets for a better understanding of plant biology. Therefore, the present work aims to examine (i) the different types of exogenous MS, (ii) how they are perceived by plants, and (iii) the plant responses and their adaptation strategies. 

## 2. Different Typologies of Mechanical Stress

Unlike other abiotic stresses, MS does not only occur due to the impact of exogenous factors. As Hamant [13] points outs, mechanical stress is both a cause for and consequence from growth heterogeneity. Indeed, mechanical cues come from both an exogenous and endogenous sources, often concurrently. Exogenous MS is induced by environmental factors (i.e., wind, heavy rains, touch, wounding, gravity alterations), while endogenous MS is a result of the plants’ own growth, cell movement, division, and morphogenesis [13,14].

As a type of mechanical force, endogenous MS has a fundamental role for both plant development and plant–environment interactions. Endogenous stress is crucial for plant growth as it directs the cells’ formation through the activity of two antagonistic parameters: the turgor pressure and the cell wall stiffness [15,16]. Cellular growth and division depend on the balance between the turgor pressure and the cell wall’s resistance to tensile strength [17]. The geometry (size and shape) of pressurized cells and the overall structure of the mechanical wall network is a key determinant of the direction of maximal tension within the cell, which can be overcome by tissue-level stresses that result from the shape or growth of the tissue [18]. This is likely to be due to the prominent role of microtubules in guiding cell division orientation [19] and their role as MS integrators [18]. These forces alter the microtubule network orientation, which has been found to dictate the direction of the cell growth by allowing for anisotropic growth, which is crucial for the plant’s morphogenesis [15,20]. In vivo studies have managed to uncover some further particularities of the impact of endogenous stress, which were recently reviewed in detail by [14,16,21]. For example, in *Arabidopsis thaliana,* the endogenous stress-related signal has been found to be overrepresented in the shoot stem cells and was dependent on ethylene signaling [22]. In poplar, endogenous ethylene produced in response to induced stem pending has been described as a stimulator of cell division in the cambial meristem [23].

Different sources of exogenous MS have diverse effects on plants, raising the question of how these environmental constraints are similar and/or different. For example, Telewski [24] grouped the exogenous mechanical stimuli impacting the stem in three groups based on how they are induced as: (i) those induced by gradients in pressure (wind in the atmosphere, currents and tides in water); (ii) those induced by gravity (such as the accumulation of ice or snow, but do not necessarily induce a gravitropic response); and (iii) those induced by touch, collectively named ‘thigmo stimuli’, which provoke thigmomorphogenesis, thigmotropism, and/thigmonasty (Box 1). This review focuses on the impact of exogenous mechanical stresses, which is briefly summarized below.

Box 1 **Glossary of general terms related to mechanical stress.**

**Mechanosensitive structure**—the location and number of mechanosensitive tissues that are involved in the response to mechanical stress [25];

**Gravitropism/Gravitropic response**—the gravity-driven growth response, which includes the perception of gravity, signal transmission, and growth response [26,27];

**Positive gravitropism**—the downward (root) growth, towards the source of gravity [27];

**Negative gravitropism**—the upward stem growth, against the source of gravity [27];

**Thigmotropism**—directional growth, which is determined by the position of the stimulus [5];

**Thigmonasty**—growth, which is not determined by the position of the stimulus [5];

**Thigmomorphogenesis**—adaptive suite of plant responses (anatomical, physiological, biochemical, biophysical, and molecular) to mechanical stress [28];

### 2.1. Wind

The ability of a plant to respond to wind (for reviews, see [29,30,31]) or waves [30,32] is strictly related to changes in their morphology and anatomy, particularly in the biomechanical properties, which enable the plant to withstand additional mechanical loading. Wind-induced sway is considered as the primary mechanical stress in terrestrial plants, triggering an interchanging compressive and tensional force, with some torsion applied in the stems and roots [25,31]. As a result of the wind applying an asymmetrical force to a plant’s side, a cantilever is created, with the rotation point in the root plate. This illustrates one of the challenges in splitting out the various forces that are at play on a plant’s structure and investigating how it reacts to these mechanical stresses [30]. This induces specialized strain-generating tissues such as reaction wood that is needed to reorient a terrestrial plant within a gravitational field [33].

### 2.2. Rain and Herbivory

Rain and animal herbivory can have a significant mechanical effect on plants, affecting their growth, integrity, reproductive success, and ability to survive. Rainfall can cause physical damage to plants through the force of the raindrops themselves, which may strip the plants of their protective outer layers, such as waxes and cuticles. Rain is also a significant source of severe plant diseases such as fungal spores and bacteria, which are transported by rain-dispersed aerosols or ballistic particles sprayed from sick plants nearby. *A. thaliana* seedlings subjected to a rain-like spray bottle were reported to accumulate jasmonic acid (JA), which promoted the expression of JA-responsive genes [34]. The mechanoresponsive *TOUCH (TCH)* genes are also known to be upregulated in response to rain-stimulating water spray [35]. Recently, trichomes, hair-like structures on the epidermis, were postulated as an early layer of the plant immune system that directly senses external mechanical forces, including raindrops, to anticipate pathogen infections in *A. thaliana* [36].

Animal and insect herbivory can also cause physical damage to plants, damaging the leaves and stems and exposing plants to infections. This physical damage can ultimately lead to altered growth and reduced ability to survive in the environment. Physical disruption and chemical elicitation are two types of herbivory defense-inducing stimuli. Physical disruption is further subdivided into wounding and mechanical stimulation (i.e., physical movement and/or vibrations), and chemical elicitation is further subdivided into substances generated from insect-associated microorganisms or from the insects themselves [37]. Upon herbivore attack detection, plants trigger various complex signal cascades (e.g., electrical and chemical signaling pathways) both locally and systemically, resulting in the activation of defense responses such as the accumulation of reactive oxygen species (ROS), Ca^2+^, defense hormones, specialized metabolites, and the release of volatile organic compounds (VOCs), which contribute to the plant’s capacity to mitigate the effects of the imposed stress [38,39]. For example, insect probing for a feeding point may damage cells along the stylet track, disrupting vital cell-to-cell interactions and releasing stored plant signals that promote mechanoresponsive gene expression [40]. Notably, above- and below-ground herbivory trigger distinct responses in terms of the hormonal and secondary metabolite responses, possibly owing to the distinct ecology of root herbivores, complex root–microbe interactions, and soil properties [41].

### 2.3. Gravity

Plant gravitropism has been studied for over 100 years, as its ubiquitous presence impacts plants’ growth redirection and development [26,42]. Gravity is sensed in specialized gravity-sensing cells known as statocytes, which convert gravity information into biochemical signals, resulting in asymmetric auxin distribution and driving asymmetric cell division/expansion (reviewed in [43]). Experiments altering gravity involve both mechanosensing and gravisensing mechanisms, making the discrimination between both perception machineries a challenging task [42]. Both MS and gravity are characterized by a similar sequence of events: (i) sensing and early gene expression, (ii) biochemical signaling formation, transduction, and feedback, and (iii) phenotypic plasticity, i.e., asymmetric organ growth [4,20,26]. Gravity induces a growth reorientation that overlaps with the plant’s response to MS, i.e., the reaction wood (RW; see Section 4.1.2) formation in stems and gravitropic curvature in horizontally stimulated roots [44], along with a higher degree of branching [45]. The root columella, which consists of polarized cells located inside the root cap, was demonstrated to be the primary site for gravity sensing and perception [46]. Numerous studies have investigated the genetic control of statocyte formation, gravity sensing, and signaling, which have been summarized in [43]. The starch-statolith hypothesis postulates that dense starch-filled organelles (statoliths) settle near the plasma membrane relative to the gravity vector within statocytes, providing directional information to the plant. The settling of statoliths initiates a biochemical cascade that promotes differential growth in the plant root or shoot elongation zones (reviewed in [47,48]). Using mathematical and kinetic tools, several models have demonstrated the intractability of gravisensing and mechanosensing, further complicating experimental designs [42]. However, similarities between the plant responses to these two environmental cues are evident and must be considered.

Distinct from gravitropism, gravity resistance is another type of plants’ response to gravity, which is evolutionarily acquired by plants following their expansion from water to land. Multiple developmental characteristics and physiological functions have been shown to be affected by hypergravity, from seed germination to cell wall composition, photosynthesis, phytohormones and secondary metabolites, oxidative stress tolerance, and plant reproductive potential (recently reviewed by Hosamani et al. [49]). Using centrifugation-induced hypergravity and microgravity conditions in space, research revealed that plants exhibit several morphological changes, including growth restriction, a short and compact body, and increased cell wall stiffness to endure gravitational stress [50]. Furthermore, an improvement in root development was observed in bread wheat under a hypergravity response, which brings the notion into agricultural relevance [51,52]. Contrasting effects in response to hypergravity and microgravity exposure were reported in plant aerial organs in terms of elongation, lateral growth, and the number of cells with transverse microtubules [53]. Nevertheless, an interaction between both gravity alteration states was proposed due to the interrelated sensing mechanisms of hypergravity and gravitropism. Hypergravity exposure has been shown to promote gravitropism in *Arabidopsis* shoots and roots through the induction of amyloplast sedimentation [54].

### 2.4. Bending, Slope, and Touch

Bending and touch are the most well-known exogenous MS, which have received significant attention due to their capacity to visibly affect plants both negatively and positively. The morphological changes induced by wind, i.e., bending, is an apparent impact of MS on plant development [13]. Because it can significantly decrease the wood economic value, it has been extensively studied in woody plant stems (see Section 4.1 on stem thigmomorphogenesis). In an extensive review, Gardiner et al. [8] discuss in-depth the various aspects of the wind–plant relationship, from the general characteristics of the wind as an external force (wind flow and load on plants) to the plant biomechanical response, i.e., the damage it causes to crops, urban, and forest trees. 

Slope is another common complex MS condition that has a significant impact on plant stability. Even though plants are known to help prevent landslides [55], little is known about the effects of the slope on the root system growth and development. Studies of four woody species (*Quercus pubescens, Q. cerris, Fraxinus ornus, Spartium junceum*) growing on slopes under natural conditions revealed morphological and architectural alterations that produced an asymmetrical root system, designated as a ‘bilateral-fan shape’ in which lateral roots developed both downslope and upslope [45,56,57]. Furthermore, plants perceive and respond to the slope early in development by changing the morphology of their root systems, modifying their biomechanical properties, and increasing their lignin content. Finally, changes in the expression levels of several genes were discovered in the roots of slope-grown plants, some of which may be homologous to genes regulating plant biomechanical properties [55]. The asymmetric root growth distribution was later confirmed in a study describing that up-slope roots were the main contributors to anchorage properties, which are characterized by more densely distributed xylem fibers [58].

The touch–response plasticity has been of interest due to its potential as a stem priming tool for yield increase in agricultural crops (both annual and perennial) and for increasing plant resistance to other types of stress that are more detrimental, e.g., herbivore insects, intensive mechanical perturbation from wind, rain, or snow, etc. [59]. Beyond the impact of direct touch, the presence of neighboring trees, e.g., the phenomenon of ‘canopy shyness’ where touching canopies of neighboring trees directs the canopy development [2], or the ‘shade avoidance syndrome’, which provides for slender stems, reduced branching, and root allocation [60], can also be considered as a form of MS. Since the mid-17th century, Darwin put forward the hypothesis where plant roots perceive touch and respond by altering their movement and growth patterns [4,11]. This has been further investigated through the primary root response to MS and especially the induction of lateral roots [61,62,63].

Bending and touch have been used as the primary methods for studying thigmomorphogenesis as they are more conveniently induced in controlled conditions. Extending on the work performed by Börnke et al. [64], who summarized some of the experimental set-ups to explore the impact of mechanical stress, we differentiate four types of treatment that are commonly used to study organ(s) and characteristic(s) of interest, i.e., flexing, vibrations (also names ‘seismic stress’), touch (rubbing or prolonged touch), and wind treatment (naturally occurring or imitated by fans) (Table 1). However, despite the body of literature regarding various types of thigmomorphogenesis, we still do not have a clear understanding of whether the different types of mechanical stimuli are perceived in the same way or how similar the plant response they evoke is.

## 3. Mechanical Stress Perception

Environmental cues are detected by a variety of mechanosensors embedded into the cell wall, membrane, or cytoskeleton. These stress sensors recognize specific stress signals, which are later converted into complex downstream signaling cascades [24]. Cell walls are particularly involved in both endogenous and exogenous MS, as plants use the mechanical properties of the cell walls for several specific functions besides mechanosensing [13]. The early sensory phase of mechanical stimulation begins shortly after contact and might persist for several hours. MS reactions are classified into the following periods: (1) the early sensory perception period and gene expression (seconds to hours), where mechanically induced changes in gene expression can occur within 5–30 min of a single stimulus and return to basal levels of expression within 1–2 h or remain altered for several hours; (2) a period of phytohormone signaling and metabolic feedback; and (3) phenotypic plasticity, which facilitates a stress acclimation phase [4].

Both the MS perception and subsequent signaling cascade involve specific upstream signals such as glutamate, external ATP, small peptides, and hormones, which are responsible for the release of secondary messengers such as calcium into the cytosol as stress-specific signatures (reviewed by Demidchik et al. [106]). Ca^2+^ ions are ubiquitous secondary messengers that translate extracellular signals into complex intracellular responses, hence playing an important role in various signal transduction pathways in both plant and animal cells [107].

### 3.1. Calcium Signaling

During the transmission of a wide range of abiotic signals, changes in cytosolic free calcium [Ca^2+^]_cyt_ are observed. Calcium signatures are decoded by calcium-binding proteins, which relay the signal for subsequent downstream signaling cascades [108]. The significance of calcium signals in plant development and environmental stress perception has recently been updated [109,110]; it shows that nuclear Ca^2+^ signaling initiates in the nucleus of *Arabidopsis* root cells and regulates primary root development, including meristem development and auxin homeostasis. The authors of the works further demonstrate that DOES NOT MAKE INFECTIONS 1 (DMI1) is essential for the nuclear Ca^2+^ signatures and primary root development (reviewed by [111,112]). In mechanically damaged *Arabidopsis*, the PLANT ELICITOR PEPTIDE 1 (PEP1) is released by the activation of METACAPSE4 (MC4) following extended high levels of [Ca^2+^]_cyt_ exclusively in the mechanically damaged cells [113]. Another study reported that wound-induced jasmonic acid biosynthesis was triggered by a calcium Ca^2+^/calmodulin (CaM)-dependent phosphorylation of a novel JAV1-JAZ8-WRKY51 (JJW) complex [114]. Calcium was found to be essential for the nuclear–cytoplasmic shuttling of *Arabidopsis* VIRE2-INTERACTING PROTEIN (VIP1) and other group I basic-region/leucine-zipper (bZIP) family proteins that interact with CaM preceding the plant response to hypo-osmotic and/or mechanical stress [115]. The mechanism of the calcium flux was remarkably reported by Bellandi et al. [116], who observed that calcium wave transmission may be described by apoplastic diffusion and the bulk flow of amino acids, which activate glutamate receptor-like (GLR) proteins as they travel through tissues. A multiparametric in vivo analysis of signaling chemicals in *Arabidopsis* was recently published using dual-reporting, transcriptionally linked, genetically encoded fluorescence indicators (2-in-1-GEFIs) [117]. The study proved that rapid cytosolic Ca^2+^ or pH changes were ABA-independent, while auxin, glutamate, ATP, PEP1, and glutathione disulfide were shown to induce cytosolic Ca^2+^, H^+^, and anion dynamics with high spatiotemporal overlapping [117]. Suda et al. recently explained the leaf closure response of the carnivorous plant *Dionaea muscipula* by linking calcium dynamics to signal memory using transgenic lines expressing the calcium sensor GCaMP6f [118]. More studies ought to focus on fully uncovering the comprehensive mechanisms underlying Ca^2+^ signal initiation, transduction, crosstalk, and propagation to predict precise downstream responses. This would allow for a better understanding of the interaction between numerous signaling systems, including Ca^2+^, reactive oxygen species (ROS), electrical, and hydraulic signals, which can help us to decipher plant stress signaling.

### 3.2. Calcium Channels

**Figure 1:**
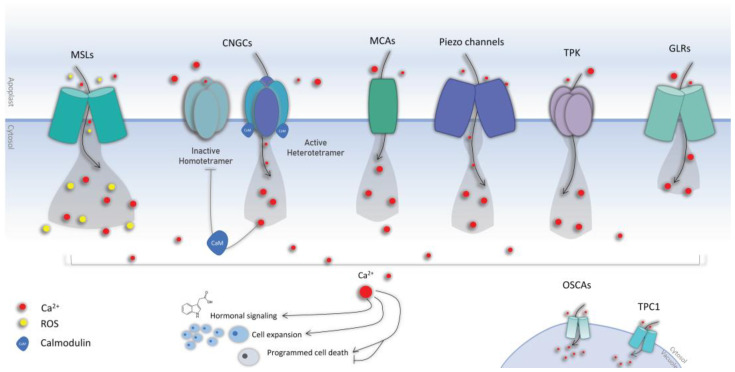
The identified mechanosensitive calcium channels. (*Left to right*) Plasma membrane-embedded mechanosensitive calcium channels include the mechanosensitive channels of small conductance-like (MSLs) and the cyclic nucleotide-gated channels (CNGCs), which may have unique functional characteristics compared with homotetrameric channels in addition to conjugating with calmodulins (CaM). Mid1-complementing activity (MCA) channels, piezo channels, the two-pore domain K^+^ (TPK) channels, and glutamate receptor-like (GLR). Vacuolar mechanosensitive calcium channels include the reduced hyperosmolarity-induced [Ca^2+^]_cyt_ increase (OSCAs) and two-pore channel (TPC1). The intracellular calcium ions regulate downstream signaling elements including hormonal signaling (ethylene, abscisic acid, and auxin).

Stress-responsive increases in [Ca^2+^]_cyt_ ion concentration are mediated by an extracellular influx or intracellular release from intracellular stores such as the vacuole, endoplasmic reticulum, or mitochondria. Calcium ions play an important function in plant downstream signaling in response to mechanical stress; they are released from intracellular reserves and readily flow into the cytoplasm when plants are subjected to mechanical stress. This calcium ion influx stimulates a variety of calcium-dependent enzymes, including calmodulin and protein kinases, which in turn activate downstream signal transduction pathways that control many cellular functions (hormone upregulation, activation of stress-responsive genes, cell expansion, programmed cell death) [119] (Figure 1). In *Arabidopsis*, 3723 genes encode for PM-localized proteins, and among them, 61 are related to Ca^2+^ transport, naturally including the putative Ca^2+^ transporters [119]. Calcium channels recognize the changes in membrane polarization in various tissues before triggering downstream signaling events in response to external stress, but also do so during developmental processes (Figure 1). Despite their central role, the identity and working mechanisms of calcium channels are not yet fully elucidated (reviewed by Koster et al. [111]). Numerous cation families were linked to calcium signaling in plants, including cyclic nucleotide-gated channels (CNGCs), ionotropic glutamate receptors, two-pore channel 1 (TPC1), annexins (ANN), and several types of mechanosensitive channels.

CNGCs are tetrameric cation channels that are activated by the cyclic nucleotides (cNMPs) adenosine 3′,5′-cyclic monophosphate (cAMP) and guanosine 3′,5′-cyclic monophosphate (cGMP). Recent research has clearly demonstrated that plant CNGCs may dynamically interact to generate both homomeric and heteromeric channels. In contrast to homotetrameric channels, there is mounting evidence that CNGCs form heterotetrameric complexes that may have distinct functional properties. These could make it easier to create stimulus-specific Ca^2+^ signatures (as monophasic, biphasic, or oscillatory increases in this second messenger in a particular cellular compartment), which could then be decoded by further downstream Ca^2+^-binding proteins that trigger a stimulus-specific adaptive response [120] (Figure 1). To date, 20 members have been identified in plants [121]. Studies have related their roles into gravitropism, pathogen defense, salt tolerance, heat and drought tolerance, root development, etc. (reviewed in [120,122]). CNGCs are predominantly present in the plasma membrane mediating the apoplast–cytosol Ca^2+^ influx, but they are also found in the endomembrane system to ensure calcium release from storage organelles. Determining the subcellular localization of individual CNGCs requires rigorous experimentation, and it remains to be determined whether CNGC distribution may be dynamic in response to cellular stimuli. CNGCs are involved in several processes such as plant nutrition, developmental signaling, abiotic stress, and immunity (reviewed in [120,122]). Here, we distill some of the recent reports unraveling the novel roles of the plant CNGC family members. CNGC2 has been linked to plant immunity and Ca^2+^ signaling due to the autoimmune phenotypes exhibited by the null mutants of CNGC2 in *Arabidopsis* [123]. Chakraborty et al. propose that the Ca^2+^ signal generated by CNGC2 is a part of the negative feedback regulation of auxin homeostasis. CNGC2 was also reported to be essential for pathogen-associated molecular patterns (PAMP)-induced Ca^2+^ signaling in *Arabidopsis*, along with CNGC4, but neither alone; CNGC2 assembles into a functional calcium channel that is blocked by calmodulin in the resting state and is phosphorylated to trigger an increase in the concentration of cytosolic calcium [124]. Additionally, CNGC2 was suggested to be a critical component in picking up the damage signal from external ATP receptors to downstream Ca^2+^ signaling in roots [125]. An ABA-mediated stomatal closure was very recently demonstrated to occur through four CNGCs, including CNGC5, 6, 9, and 12 [126]. Another report identified and characterized CNGC5, CNGC6, and CNGC9 as Ca^2+^ channels involved in auxin signaling, which is essential for root hair growth in *Arabidopsis*, with different roles in root hairs being provided by the conditional player CNGC14 [127]. CNGC14 is a mediator of rapid auxin- and gravity-induced Ca^2+^ signaling the roots of *A. thaliana* [128]; it has also been linked to Ca^2+^ influx in *Arabidopsis* root hairs. A study showed that *cngc14* mutants lacked an auxin-triggered Ca^2+^ as well as an AUX1-mediated H^+^-influx, supporting the model in which it acts as the bona fide auxin-activated Ca^2+^-permeable channel at the plasma membrane [129]. CNGCs are directly controlled by the conserved Ca^2+^ sensor calmodulin (CaM), with one or more CaM-binding domains (CaMBDs) found in both the cytosolic and N-terminus of all CNGCs [130].

Calmodulins (CaMs) are a primary group of well-characterized Ca^2+^ sensors that are ubiquitously present in eukaryotes. After Ca^2+^ binding, CaMs display conformational changes that facilitate their interaction with downstream target proteins [114]. They have been linked to the regulation of metal ions uptake, generation of reactive oxygen species, and modulation of transcription factors that are involved in various pathways [131]. Recent studies identified CaM7-CNGC14 as a novel interacting module that regulates polar growth in root hairs by controlling the tip-focused Ca^2+^ signal [132,133]. Using the *Xenopus laevis* oocyte heterologous expression system, a recent report discerned the calcium channel activity of two CNGCs, CNGC11 and CNGC12, by using the two-electrode voltage-clamp technique [134]. The study concluded that CNGC12, but not CNGC11, functions as an active calcium channel, whose activity was significantly enhanced when co-expressed with calmodulin1 (CaM1). In fact, recent advances identified calmodulins as CNGC molecular switches that bind to their calmodulin-binding domains. Recent research has confirmed a model of CNGC regulation mediated by CaMs in which a CNGC domain is permanently anchored by a calcium-free calmodulin. Through this interaction, calmodulin can accurately detect Ca^2+^ in the channel complex and provide Ca^2+^-dependent feedback [135] (Figure 1). Meena et al. [136] reports a key mechanistic role for the Ca^2+^ channel CNGC19 in the recognition of herbivory and activation of defense signaling following interaction with the herbivory-specific calmodulin 2 (CaM2) in *Arabidopsis* [137]. Under low calcium levels, calcium-free CaM2 was shown to interact with the CNGC18/8 complex, activating the calcium influx channel. Once cytosolic calcium levels are increased, the calcium bound CaM2 dissociates from the CNGC18/8 heterotetramer, closing the channel and initiating a decrease in cellular calcium levels [137]. Despite the recent advances in understanding the roles and structures of plant CNGCs, knowledge gaps have yet to be addressed, including the selectivity of these channels, their subcellular localization, and the structure of their complexes in vivo, which remain unanswered.

Unlike other calcium channels, hyperosmolality-gated calcium-permeable channels (OSCAs) was only recently identified [138]. OSCAs have nine transmembrane helices with a short extracellular N-terminus and larger C-terminus; they constitute a 15-member family in *Arabidopsis* [138]. An in silico analysis revealed that OSCAs are an evolutionarily conserved family of mechanosensitive Ca^2+^-permeable cation channels [139,140]. In *Arabidopsis*, OSCA1.2 was demonstrated to be an inherently mechanosensitive, pore-forming calcium channel with membrane tension activation characteristics [139].

### 3.3. Mechanosensitive Channels

Mechanically activated (or mechanosensitive) ion channels (MSCs) are membrane-bound proteins in eucaryotes that sense membrane tension and mechanical osmotic stimuli, converting it to electrical signals that trigger downstream signaling cascades [20,141]. Plant MSCs include the families of the mid1-complementing activity (MCA) channels [142,143], the mechanosensitive channel of small conductance-like (MSL), reduced hyperosmolarity-induced calcium increase (OSCA), and the piezo channel families (Figure 1).

#### 3.3.1. The Mid1-Complementing Activity (MCA) Channels

MCAs are plant-specific mechanosensitive ion channels distinguished by a single transmembrane domain. In *Arabidopsis*, they were shown to take part in the root perception of external mechanical stresses [144]. The overexpression of MCA1 and MCA2 resulted in higher Ca^2+^ uptake in response to hypo-osmotic shock [145]. MCA1 was reported to meditate ROS accumulation in synergy with another mechanosensitive channel, MSL10 [146]. MCA1 is also required for cell wall integrity signaling [147]. Okamoto et al. [148] recently identified three essential components of the *Arabidopsis* mechanotransduction pathway, namely MCA1, the ethylene-regulated microtubule-associated protein WDL5, and a versatile co-receptor BRI1-associated receptor kinase 1 (BAK1) belonging to the receptor-like kinases superfamily (RLKs). Temperature-dependent calcium influx into the cytosol was also reported to be mediated through MCA1 and MCA2 [119,149]. The same genes were also linked to the perception of gravity signals in the *Arabidopsis* hypocotyl, suggesting a pivotal role in the resistance to hypergravity [150].

#### 3.3.2. The Mechanosensitive Channel of Small Conductance-like (MSL)

The mechanosensitive channels of small conductance-like (MSL) are non-selective ion channels that are directly gated by membrane tension and found throughout bacteria, archaea, some fungi, algae, and plants [151]. The *Arabidopsis* MSL9 and MSL10 are essential for mechanosensitive channel activity in the plasma membrane of root cells [152]. MSL10 was shown to play two genetically separable functions with involvement in both mechanoreception and ROS-mediated cell death [153]. A recent study reported that the N-terminus phosphorylation of MSL10 was mandatory for cell swelling-induced programmed cell death in addition to other hypo-osmotic shock responses in *Arabidopsis* seedlings, including a cytoplasmic calcium transient within the first few seconds, accumulation of ROS within the first 30 min, and increased transcript levels of mechano-inducible genes within 60 min [146]. More recently, another study reported that the stretch-activated MSL10 plays a pivotal role in wound-induced electrical signals in *Arabidopsis* distal leaves as well as the amplitude and kinetics of the systemic Ca^2+^ wave [154]. The study concluded that MSL10 is part of the glutamate receptor-like proteins (GLRs), linking the mechano-sensing, ion fluxes, membrane depolarization, and propagation of electrical signals. Tran et al. [155] suggested that MSL10 might represent a system of oscillatory perception in plants, which acts as both a classical transducer of sustained force and as a transducer of mechanical oscillations. MSLs were proven to play pivotal roles in the rapid wound-induced plant signaling cascade. Toyota et al. reported that the local administration of glutamate can cause both systemic propagation of a Ca^2+^ elevation and activation of JA defensive responses throughout the plant in a GLR3.3- and GLR3.6-dependent manner [156]. In fact, GLRs are responsible for the Ca^2+^ fluxes involved in the transmission of systemic signals, which detect the release of glutamate from the apoplast as an early trigger for systemic wound responses [156].

#### 3.3.3. Piezo

The piezo sensors are cell membrane mechanical signal transducers first identified in animal cells. In plants, they are plasma membrane-localized cation channels involved in diverse mechanosensory processes. In *Arabidopsis*, piezo was shown to regulate the spread of viruses [157]. Piezo was observed to be mainly expressed in the root cap, but also in guard cells, vascular tissue, and pollen [158]. Owing to their role in the Ca^2+^ transportation upon mechanical stimuli, it stands as an important player in the mechanical stress perception mechanism [158]. Genetic analysis confirmed that the same gene plays an important role in root mechanotransduction, confirming that piezo are physiologically relevant mechanosensitive ion channels across the animal and plant kingdoms [159]. Radin et al. [160] observed that the *Arabidopsis* Piezo1 and Piezo2 were tonoplast-localized with an essential function for the vacuole tubulation in the tips of pollen tubes. Authors attribute this subcellular localization to the relatively higher mobility of the tonoplast compared with the plasma membrane, rendering it a more effective location for mechanosensory proteins [160].

#### 3.3.4. Other Channels

Further candidates with mechanosensitive and calcium channeling activity are continuously emerging. For instance, cell wall integrity sensors such as the receptor-like kinases (RLKs), *Catharanthus roseus* RLK (CrRLK1L), Theseus1, Feronia, and the wall-associated kinases could be involved in the machinery of mechanical stress perception by transducing stimuli occurring in the cell wall [143]; however, it remains to be understood whether they act in synergy with or independently from the mechanosensitive ion channels. The mitogen-activated protein kinase kinase (MKK1 and MKK2) were recently linked to thigmomorphogenesis following a double knockout characterization in response to machine-driven hair-induced touch stimulation [161]. MILDEW RESISTANCE LOCUS O4 (MLO4) was recently described as a typical Ca^2+^ channel that links touch stimulation to Ca^2+^ elevation in root tip cells [162]. Zhang et al. reported that the *mlo4* mutant was defective in root mechanosensing and displayed a hardly detectable post-barrier exposure calcium spike [162]. The *Arabidopsis* annexin 1 (ANN1)—a soluble protein lacking transmembrane helices—was identified as a positive regulator of local and systemic Ca^2+^ responses following mechanical wounding [163]. Microtubules have a key role in plant morphogenesis; the contribution of mechanical stress in guiding *microtubule* behavior was synthesized by [15], which presented a model explaining microtubule dynamics and their reorganization capacity as well as a synthetic depiction of the alleged mechanotransducers at work. Using a cytoskeleton-targeting pharmacological approach in combination with mechanical stimulation, Shevchenko et al. demonstrated the effects of mechanical perturbation on cytoskeleton regulation in *Arabidopsis* seedling roots [81]. The authors claim that cortical microtubules (cMTs) play a leading role in plant cell mechanosensing along with microtubule-associated proteins 65 (MAP65-1), cytoplasmic linker-associated protein (CLASP), and formins (FH1/FH4). Furthermore, they demonstrated that slow clinorotation—a rotation about an axis at so slow a rate that the centrifugal force is so small as to be discounted—was able to induce a MS response [81].

The currently identified mechanosensitive channels are unlikely to explain the integrity of the observed mechanosensitive activities [142]. Discovering new mechanosensitive channels would require the combination of in silico homology-based screens with functional in vivo investigations. Future research ought to consider both the stem and roots, as specific genetic and physiological differences in response to MS was observed between these two compartments. New technologies such as optogenetics and fluorescent imaging can also be used to identify and characterize mechanosensitive channels in plants. Optogenetics combines genetic engineering with optical control to study the ion channels in plants, while fluorescent imaging allows the visualization of the location of these channels within the cell.

## 4. The Plant Response to Mechanical Stress

**Figure 2:**
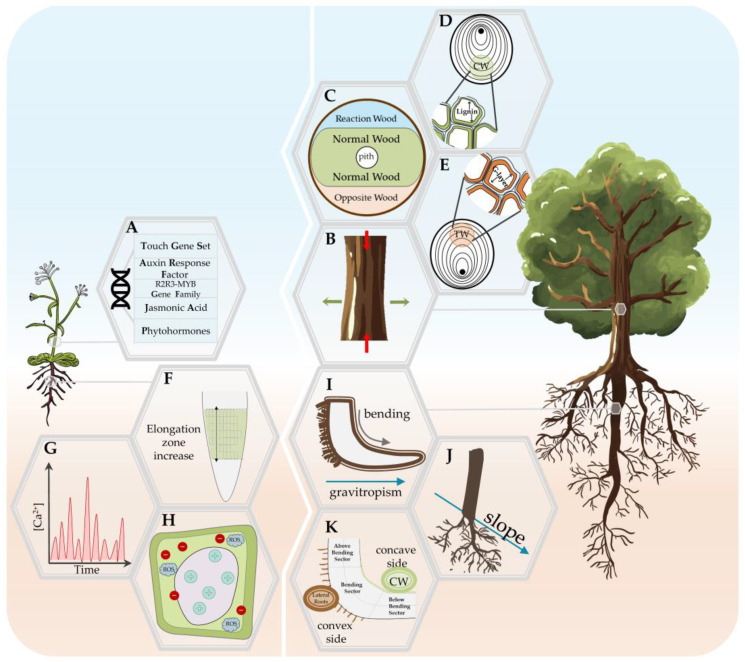
Summary of the mechanical stress response in annual (*Arabidopsis*; left panel) and woody plants (right panel). (**A**) Mechanical stress-related molecular responses in *Arabidopsis* stem. (**B**) Zones of differentiated growth in woody plant stems subjected to mechanical stress (reaction wood, RW; normal wood, NW; opposite wood, OW). (**C**) Location and characteristics of the tension wood (TW) formation, i.e., the RW in the stem of the Angiosperm specie. (**D**) Location and characteristics of the compression wood (CW) formation, i.e., the RW in the stem of the Gymnosperm specie. (**E**) Reduction in the elongation growth and increase in the radial thickness in the stem of woody plants. (**F**) Increase in the elongation zone with radially symmetric changes in cell expansion and elongation in the root of *Arabidopsis*. (**G**) Stimulus-specific rapid and transient increase in cytosolic calcium in *Arabidopsis* root. (**H**) Apoplastic alkalinization, cytoplasmic acidification, and the production of apoplastic reactive oxygen species (ROS). (**I**) Lateral root initiation as a response to either gravitropic curvature or manual woody root bending. (**J**) ‘Bilateral-fan shape’ lateral roots root distribution in slope conditions. (**K**) Asymmetric response of three root bending sectors on the concave and convex side of bent woody roots.

The aforementioned channels evoke various responses across the plant organs, notably on a morphological level, and are preceded by a range of biochemical changes. Undoubtably, these responses depend on the type of MS as well as the developmental stage of the plant and species biological characteristics (e.g., see [23,78]). Table 1 summarizes the variables from in vivo studies that have provided insights into the plant response to MS. Furthermore, in the following paragraphs, we outline the common thigmomorphogenetic characteristics of the stem and root; we note that, to the best of our knowledge and as other authors have shown, e.g., [83], there is no universal response to MS in woody and annual plants (Figure 2).

### 4.1. Thigmomorphogenesis in Stem

As Braam [164] reviewed, thigmomorphogenesis impacts plant species differently, and the touch stimuli can trigger different responses in the plant above-ground organs, i.e., leaves in carnivorous plants, modified leaves/stems in climbing plants, flowers in some species where self-pollination is possible, etc. The most iconic illustration of this is the thigmomorphogenetic response of *Mimosa pudica*, also referred to as ‘touch me not’. Mediating a motor organ named pulvinus, the plant leaflets rapidly fold in response to exogenous MS, using a long-distance rapid electrical signal [165] and calcium fluxes [166]. Our review focuses on the response in erected plants and thus on the stem response, especially through the particularities of the response found in *Arabidopsis* as a model representative of annual plants and through the reaction wood formation (RW) in perennial plants (trees). However, as Liu et al. [60] pointed out, there are considerable differences regarding the stoloniferous and stem of aquatic plants as an additional aspect in terms of plants thigmomorphogenesis.

#### 4.1.1. Mechanical Stress Response in Annual Plants: *Arabidopsis* Model

In response to mechanical stimuli such as wind or touch, stems undergo physiological and developmental changes that enhance resistance to subsequent MS (Figure 2A). In general, plants that are grown in windy environments are shorter, stockier, and often have altered flexibility [167,168].

In *Arabidopsis*, as a rosette plant, the ontogenetical function of the stem is different from the perennial counterpart, in which it contributes for long-term stability, structural and mechanical fitness, and where the aforementioned services are not prioritized [85,169,170]. However, the biological characteristics of *Arabidopsis,* i.e., the possibility to induce a stem in the secondary structure by decapitation, by reducing light exposure (‘short-day conditions’), or by increasing the weight load of the stem [164,171], along with the knowledge/data availability regarding the wide scope of the species’ physio-molecular process have been exploited in terms of studying the mechanisms of the stem response to MS. Wind stimulation has been shown to proportionally impact the degree of branching and basal fruit production in *Arabidopsis* plants [59].

The serendipitous discovery of the *Arabidopsis* touch (TCH) gene set has spiked an interest in the thigmomorphogenetic molecular mechanisms [83,92]. The roles played by the TCH gene family is not limited to MS as they were associated with upregulation via exogenous auxin and/or brassinosteroid and the fluctuation of free cytosolic calcium ion (Ca^2+^) as a secondary messenger in a variety of signal transduction pathways [92], opening new research channels. The generally observed stem thickness increase in response to MS does not always occur in *Arabidopsis*, but through the application of weight on the stem, a type of compression force can induce the formation of cambium-like tissues [83]. Auxin was found to support the secondary xylem formation, and three auxin response factor (ARF) genes (ARF2, ARF4, and ARF12) are assumed to play a particularly significant role during the wood formation [30]. From the previously mentioned R2R3-MYB gene family, four MYB transcription factors are considered as candidate regulatory genes for wood formation, and three of them (*AtMYB77, AtMYB73, AtMYB44)* seem to have similar functions in stem development [30]. Some of the TCH genes have additionally been linked to jasmonate signaling [13]. Mechanostimulation involves jasmonic acid (JA) signaling pathways as part of the cambium regulation, which induces the JA production and expression of JA biosynthesis genes [172] and is required for the wound-induced growth-regulation [173]. Katanin-dependent microtubule dynamics were found to increase the cell competence to respond to MS by enhancing the cells’ ability to adapt to their growth according to the neighbors [174]. The role of ethylene, auxin, cytokinins, and gibberellins in the vascular development of *Arabidopsis* has also been confirmed [175].

#### 4.1.2. Woody Plants’ Stem Response—The Role of Reaction Wood

The perennial habit is associated with a wide range of morphological and physiological traits that are likely necessitated by the greater range of environmental and seasonal cues encountered by these plants compared with their annual counterparts [176]. In the stem of perennial woody plants, mechanical stress induced a reduction in elongation growth while increasing the radial thickness, i.e., reduced height and increased diameter, respectively [86,164], having a visible and direct impact on the yield/biomass production (Figure 2B). The objective of the woody plants thigmomorphogenetic response is non-vertical axis reorientation, which is achieved through the RW formation at points where the force (compression or tension) can push the stem towards its original position [177]. This response occurs due to the heterogeneity of the cambial region activity, and it mainly involves the wood, also called secondary xylem, which ensures the mechanical support and long-distance conductivity of water and nutrients [178,179,180]. Wood is naturally composed of cellulose microfibrils situated in the hemicelluloses and lignin matrix which, under load and over time, exhibit anatomical and chemical deformations [181]. These changes can reduce the wood value from an economical aspect, which has encouraged a significant body of literature to address the stem response to MS [182]. MS is also considered necessary for the differentiation of xylem cells, but the exact mechanisms of its impact are not clear [180,183]. Once a stem is bent, the asymmetrical response is exhibited as a formation of RW and opposite wood (OW) (Box 2; Figure 2C). The location and characteristics of RW differ between gymnosperms and angiosperms, respectively, and compression wood (CW) and tension wood (TW) (Box 2), and they further impact the hydraulic and mechanical wood properties through the changes in the wood properties [28,180]. Variations in both CW and TW appear due to the species characteristics and age, environmental conditions, stress type, and compression severity [177,178,179,180,181,182]. However, both employ similar basic mechanisms for sensing the stress stimulus and thigmomorphogenetic response, which differs in structural and mechanical context [177] and will be briefly summarized in the following paragraphs (Figure 2D,E).

The CW forms on the lower (concave) compressed side of the bent stem/branch in gymnosperm perennials (Figure 2D), and its main function is to push back the leaning stem to an upright position by compression stress [184]. The changes associated with CW are one of main contributors to reduced wood quality and fiber products [185], which is why we have significant knowledge on its anatomical and chemical features. The compression induces longitudinal shrinkage in comparison to normal wood (NW), which is related to a larger microfibril angle and increased lignification, both of which have been used to investigate the wood mechanical behavior and contribute to the lower stiffness of CW [181,182,186]. Another anatomical particularity of CW is shortened tracheids compared with NW and OW from the same tree, with changes in the shape and deformation of the tips [179,182]. In addition to higher lignin, the CW also contains higher amounts of (1-4)-β-galactan and lower amounts of cellulose, mannan, and xylan [182], an inverse correlation that we can observe again in the case of TW.

Box 2 **Glossary of terms related to woody structure response to mechanical stress.**

**Reaction wood (RW)**—natural response of woody plants to mechanical stress via asymmetrical formation of secondary xylem tissue, aimed to reinforce the structure and redirect the growth [27,28];

**Normal wood (NW)**—wood formed in the absence of stimulus [28];

**Flexure wood (FW)**—specific wood produced by the vascular cambium in trees growing in a windy environment, characterized with increased secondary xylem production and decreased elastic modulus in comparison to normal wood [28];

**Compression wood (CW)**—RW in Gymnosperm formed on the lower side of inclined stems or branches, characterized by a high lignin and low cellulose composition due to the generation of a compressive force to push the stem up [27,28]. Tissue with the same characteristics has been noted on the lower (concave) side in bent poplar root [28,187];

**Tension wood (TW)**—RW in dicotyledonous Angiosperm formed on the upper side of leaning stems or branches when the reorientation process being, characterized by a low lignin and high cellulose composition [27,28];

**Opposite wood (OW)**—part of the asymmetrical response to mechanical stress, located opposite the RW characterized by properties intermediate between NW and RW [28,187];

The TW forms on the upper (convex) stretched side of the bent stem/branch in angiosperm perennials (Figure 2E). Similar to CW, across species, TW exhibits a wide range of organizational variations (see comprehensive review in [188]), but its main anatomical characteristic are the G-fibers, which are xylem fibers with a smaller radial diameter that have an additional thick layer on the inner side of the secondary wall and which form an additional layer with a translucent gelatinous appearance, which is not very cohesive with the rest of the cell wall layers, named the G-layer [178,179]. The G-layer is mainly completely composed of cellulose, but the presence of lignin, xyloglucans and xyloglucan-synthesising proteins, pectins, and rhamnogalacturonan I, arabinogalactan, and arabinogalactan proteins has been confirmed [177,179]. However, as the detected lignin content has been minimal or non-existing, TW is generally considered to have increased cellulose and reduced lignin content [177,178] as the other side of the previously mentioned negative correlation. The G-layer is further characterized by a higher porosity, allowing for higher water content, which is assumed to be the reason for the gelatinous appearance and capacity of the G-layer for transversal swelling/shrinking [177]. The TW formation results from an increased cell division rate, i.e., cambial activity [179].

The role of phytohormones in the thigmomorphogenetic response and their involvement in CW and TW formation have been acknowledged and studied, but the results and conclusions are not consistent and are difficult to compare (for a detailed review, see [182] for CW and [177] for TW). This is not only due to the general involvement of the phytohormones in many aspects of the plant development, but also due to the combined effect that hormones have with each other and with other parts of the stress regulation mechanism. To briefly summarize, it appears that CW formation mainly involves auxin and ethylene along with reduced endogenous cytokinins and abscisic acid [33,182]. In TW formation, while the role of ethylene has been continuously confirmed, the role of auxin is not yet clearly defined but appears to be a crucial part for RW formation [177,178]. More recently, cytokinins and brassinosteroids were also associated with TW formation by [28] and [27], respectively. Gibberellin’s role in CW formation has been dismissed, but there has been some evidence for its role in TW formation, where it has been shown to be able to induce cambial growth and G-fibers differentiation [27,177,189].

Previous research has largely focused on the anatomical and morphological characteristics of RW. The same attention has not been given to the molecular mechanisms of secondary growth [171], and the current interest is indeed focused on the molecular and signaling aspects of RW formation. However, there has been no consensus whether a model species comparison is a suitable approach between *Arabidopsis* and perennials, as well as different perennial species. While key regulators have been observed when it comes to the secondary development for both herbaceous and woody plants [175], the issue remains that RW formation does not naturally occur in herbaceous model species such as *Arabidopsis* [7]. Using poplars as a model species does help overcome some difficulties, but the shortfall regarding molecular studies in perennial species and, in gymnosperms even more so, remains [7,190]. In CW, gene expression analysis uncovered the upregulation of genes involved in the gravitropic response of the stem, i.e., lignin biosynthesis, ethylene forming enzymes, and cell wall proteins (biosynthetic enzymes, carbohydrate metabolism and regulatory proteins, monolignol biosynthesis, arabinogalactan, and proline-rich proteins) [182,185]. A particular point of interest is the R2R3-MYB family, which regulates the lignin and phenylpropanoid metabolism during wood formation and whose involvement has been confirmed in conifers [190]. Pilate et al. [7] provided a comprehensive review regarding TW genomic studies, indicating the potential of TW to provide a better understanding of the molecular mechanisms of wood formation and their properties. More recently, studies focusing on the early changes in the poplar transcriptome have contributed to a better understanding of the thigmomorphogenesis. Pomiès et al. [69] have investigated the response due to single or repeated bending, concluding that while major gene expression changes take place in the first two hours post-bending, there are several mechanistic pathways involved in the response, starting from the genes involved in the general response to abiotic stress (ROS, Ca^2+^, and jasmonic acid signalling) to more specific genes involved in cell wall and wood development. Using an innovative isotropic device, Lopez et al. [27] have managed to isolate the early (30 min) molecular response to gravistimulation, confirming again the activity in the cell wall and wood formation and noting on about 200 xylem-regulated genes that have not yet been functionally characterized.

### 4.2. Thigmomorphogenesis in the Roots

The obstacles encountered by roots during soil penetration invariably cause thigmomorphogenesis [13]. Root ecology research faces numerous challenges, ranging from the biological characteristics of the organ to the design of relevant experimental studies [191]. These difficulties are even more accentuated in the case of root thigmomorphogenesis due to the previously mentioned factors of MS variability in experimental design (Table 1) and sampling difficulties. As with the stem, in the following paragraphs, we summarize the currently available knowledge regarding woody and young plants roots (Figure 2) with the related characteristic that generally separates dicotyledonous from monocotyledonous plants, respectively. As the plant matures, the primary roots of dicots develop a secondary developing additional xylem and phloem to support the expanding root and shoot system.

#### 4.2.1. Young Roots Response to Mechanical Stress

The impact of mechanical stimuli on primary roots induces changes in the growth direction that can alter the lateral root (LR) location, with the LRs emanating from the convex side of the arising curves rather than being in a preset distribution [98,192,193,194,195]. The cascade of events leading to LR formation from the xylem pericycle cells has been well-studied in *Arabidopsis,* which is shown to be strictly related to a transient spatio-temporal accumulation of auxins along the parental root axis [196,197]. In particular, Ditengou et al. [98] observed a delocalization of the auxin carrier PIN1 in a single protoxylem cell, followed by auxin accumulation at the site of lateral root induction. Another study confirmed the LR emission on the convex side of the bending in a timepoint as short as 20 s of transient bend [61]. LR emission has also been linked to calcium signaling, which translates the mechanical forces to a developmental response in the roots [61]. Because plants detect mechanical stimuli to identify neighboring barriers and alter their growth patterns to acclimate to their surroundings, another commonly used approach in MS studies is root barrier exposure. External and endogenously generated mechanical forces consistently cause stimulus-specific, rapid, and transient increases in cytosolic Ca^2+^ [142]. Barrier exposure was shown to trigger an apoplastic alkalinization, cytoplasmic acidification, and the production of apoplastic reactive oxygen species (ROS) [198]. Jacobsen et al. [63] used an in vitro barrier system analysis to study the *Arabidopsis* root response to short-term mechanical impedance (up to 30 h) through global transcriptional profiling. The results uncovered radially asymmetric changes in cell expansion and elongation and reduced root length in addition to a shorter distance of root hair emergence from the root tip (Figure 2F). The ROS, signaling genes linked to ethylene and auxin differential gradients, and transcriptional activation of ROS were all part of the early response of *Arabidopsis* roots [63]. The role of auxin in thigmotropism during plant–obstacle interactions has recently been established, where it was reported that PIN-FORMED (PIN)-mediated polar auxin transport enables root bending prior to obstacle avoidance [99]. Some authors suggest that in *Arabidopsis*, tension forces acting in the convex side of bent root induces an increase in Ca^2+^ levels in specific pericycle cells, becoming a ‘founder cell’ of a new lateral root [61,199] (Figure 2G). This Ca^2+^ increase leads to: (a) an alteration in ROS and cytosolic acidification, which is known to elicit signaling events; and (b) a cell wall alkalinization, known to rigidify the cell wall matrix (Figure 2E). Diaz-Sala [200] suggested that mechanosensitive ion channels that are present on the plasma membranes could generate electric action potentials that propagate on a short distance from cell to cell along with the plasma membrane network and through plasmodesmata (or alternatively through phloem cells over a longer distance) inducing modifications in the cell walls, creating specific interactions between the cell wall and cytoskeleton and alterations of the microtubule dynamics.

#### 4.2.2. Woody Roots Response

In woody plants, very few studies are available with respect to the anatomical, morphological, biochemical, and molecular aspects of woody roots’ response to slope or bending stress [28,95,187,201] (Figure 2I). As reported in Section 2.4, woody root growth on natural slope conditions produced an asymmetrical root system, designated as a ‘bilateral-fan shape’, in which lateral roots developed both downslope and upslope [34,39,40] (Figure 2J). However, mechanical constraints do not induce the same type of response in the stem and roots. Furthermore, roots subjected to similar mechanical constraints that are imposed to the stem may develop extremely dissimilar RW. Indeed, in poplar plants, bending induces TW formation on the ‘upper’ convex stretched side of the stem or branch, whereas a CW similar to gymnosperm stems is formed in the ‘lower’ concave compressed side after bending. Hellgren et al. [202] found that the formation of TW in poplar are not mediated by changes in the indole-3-acetic acid (IAA) level in the cambial tissues. On the contrary, a higher amount of endogenous IAA was detected at the side of the cambial region-forming CW, which could act as a spatial regulator of cambial activity, enhancing the cell division rate and conferring key positional information to the cells of the cambial zone’s surrounding tissues for differentiation/RW initiation [187,203,204].

The woody roots’ MS response was shown to be temporally and spatially modulated by an intricate interplay of different signal transduction pathways, involving reactive oxygen species (ROS), hormones (indole acetic acid, gibberellins, ABA, and ethylene), and specific molecular factors regulating lignin deposition, cell wall integrity, and lateral root formation [201,205,206,207,208]. Trupiano et al. [201] postulated a mechanical force distribution model where the convex and concave sides of each bent root sector are subjected to different mechanical force distributions, with the tension forces applied to the convex side and the compression forces concentrated on the concave side. Side- and sector-specific strategies were used by bent roots to maintain water uptake and transport in a deforming condition that was induced by tension and compression forces; this resulted in an increased xylem thickness on the compressed side and enhanced lateral root formation at the tension site (Figure 2K).

Following a 6-month root bending stress test, the woody roots of *Populus nigra* displayed a reaction wood (RW) formation due to compression forces at the concave side [187], showing root-specific characteristics in comparison to those produced in the bent stem. The woody roots’ RW is characterized by a low vessel density and high lignin content mainly triggered by auxin, and it is associated with the induction of cambium cell activity [187]. The research also provides some initial understanding of the mechanisms controlling this compression-induced wood on the concave side, characterized by the activation of specific proteins that govern cell wall deformation, lignification, and xylem differentiation [187]. A similar study applying a shorter (2 months) root bending stress test observed a bending sector-specific distribution of phytohormones (auxin, cytokinin, and abscisic acid) that reflected adaptations to compression- or tension-specific forces [209]. These changes were later confirmed in the mechanics of *Arabidopsis* seedling emergence. A recent study proposed a model for explaining the hypocotyl bending mechanics [97], reporting that auxin maxima are generated on the inner side of the bent by polar auxin transport through the auxin transport machinery components PIN3, PIN4, PIN7, and AUX1, promoting pectin with a high degree of methylesterification and therefore stiffening the wall and leading to a slower rate of cell elongation; this is in opposition to the outer side, which had low auxin levels favoring pectin demethylesterification, cell wall loosening, and faster cell elongation [97]. In another study on bent woody roots of poplar plants, De Zio et al. [28] observed the asymmetrical sector-specific response. These differences are expressed across measured parameters, with a higher lignin concentration in the above bending sector (ABS) and lower amount of carbohydrates on the concave side of the ABS, as well as a reduced amount of indole acetic acid (IAA) in the convex side of both the bending (BS) and below bending sector (BBS), and RW formation due to increased cambial cell activity on the concave side of the BS and BBS [28]. These changes were found to be strictly correlated to the ability of the vascular cambium cells to perceive specific signals and, in turn, to orchestrate specific genes leading to RW (towards the concave side) or lateral roots (towards the convex side) formation. Recently, Dimitrova et al. [210] provided novel information regarding the response coordination, communication, and potential signaling pathways that were asymmetrically activated along the main root axis, which were mainly delegated to Ca^2+^ (for new lateral root formation) and ROS (for gravitropic response and lignin accumulation) signatures. Furthermore, some of the data indicate that the concave side of the bent sector, where the mechanical forces are the most intense, communicates to the other (neighbor and distant) sectors, inducing spatially related strategies to ensure water uptake and accompanying cell modification [210]. The communication between these portions is supposed to engage in short distance signals, such as chemical and electrical signaling, plasma membrane hydraulic pulses, or plasmodesmata and meristematic connectomes [211], to cover long distances and adjust the root body to its surrounding environment.

During the past few decades, research efforts have provided a partial understanding of the response to MS in plant roots. However, large gaps remain, especially regarding the specific physiological, molecular, and genetic processes involved in mechanosensing and mechanotransduction. Thus, there remains a need for future technologically advanced research that is focused on the early events of the woody root bending response, as this would help in the understanding of plant tissues organization along with cell-to-cell communication between neighboring and distant cells.

## 5. Concluding Remarks

Mechanosensing and mechanotransduction are key biological phenomena that allow plants to perceive and respond in a well-coordinated manner to mechanical stimuli. The spatio-temporal patterns of the thigmomorphogenetic growth response following mechanical stimuli perception are highly variable among annual and perennial plant species and among different organs (root, stem, and leaf). Although many details of mechanically induced plant responses have emerged over the past decades, there is still much to be understood about the effects of MS on plants. For instance, the precise physiological and molecular mechanisms behind plant adaptation to mechanical stresses remain elusive. Unraveling the underlying mechanisms of surface-sensing and downstream signaling pathways is necessary to decipher how tension and compression forces are differently sensed and transmitted in the stems and roots of annual and perennial plants. The key environmental factors that influence the response of plants to mechanical stress have also yet to be determined. Furthermore, how mechanical stress affects the growth, development, and crop productivity is another important aspect to be addressed. Future research exploiting new technological tools, e.g., single-cell analysis coupled with fine-tuned modeling and in silico approaches, could produce valuable knowledge about the physiological and/or molecular markers of the early recognition of mechanical stress, further elucidating the complex interplay between signals and responses that involve downstream effects, effectors, changes in cell adhesion, and communication properties.

A comprehensive knowledge of plant mechanical stress responses should have significant commercial potential to generate novel plant-based materials for engineering and construction applications, such as biocomposites and biodegradable polymers. Furthermore, in some economic growth models where industry builds on the expanse of farmland regions, which has been exacerbated by global climate change that has shrunk accessible agricultural lands, it may be required to cultivate plants on unfavorable sites with barren, dry, or rocky soils. As a result, scientists may apply this knowledge to design better methods for safeguarding trees and promoting plant growth and development on these lands or for developing more robust plants that are able to tolerate strong winds, hail, and/or mechanical damage from agricultural machines. This might lead to improved quality and productivity of crops and enhanced food security.

## Figures and Tables

**Table 1:** Summary of experimental designs with the different methods of mechanical stress applications to various plants species and organs of interest.

MS	Method/Duration	Species	Organ	Observations	**Reference**
**Bending**	Bending device/ Transient—5 months	*Populus* sp.	Stem	RW formation on the convex side	[28,65,66,67,68,69,70]
	Lead sheet compression/2 or 7 days	*Arabidopsis thaliana*		FLA11 and FLA12 are possible MS-responsive cell surface sensors regulating stem secondary wall development	[71]
	N/A/4–40 h	*Populus tremuloides*		Understanding of *CesA* cDNA (*PtCesA*) regulation in RW formation	[72]
	Paper-mediated/Daily, 4 months	*Psammochloa villosa*		Decreased plant height, total biomass, and root/shoot ratio	[73]
	Plastic tube pressed on the stem base/5 days	*Caesalpiniaceae/* *Clusiaceae*		Variable responses between five examined species	[74]
	Manual bending/1 week	*Acacia koa*		Reduced stem elongation, increased stem diameter, increase of anthocyanin and lignin.	[75]
**Bending/flame**	Manual/8 s	*Populus tremula x alba*		Inhibited primary growth, JA-mediated response	[76]
**Bending**	Clamping rings/transient			Extracellular electrical signaling	[77]
**Gravistimulation**	Tilting/24 h			Identification of key genes regulated in the early gravitropic response	[27]
**Wounding**	Forceps/transient	*Helianthus annus*	Hypocotyl	identification of GSNOT and SNO as key new elements in the wound signaling pathway	[78]
	Bark removal with saw and chisel	*Populus* sp.	Stem	Increased wall thickness, modified lignin topochemistry	[79]
	With hemostat/transient	*Arabidopsis thaliana*	Leaves	Identification of rapid wound-responsive genes	[80]
**Raindrop**	Droplets/15 min	*Arabidopsis thaliana*	Leaves	Intercellular calcium waves, induction of defence-related genes	[36]
**Brush**	Brush/≤60 min				
**Clinorotation**	Clinorotation/2 days	*Arabidopsis thaliana*	Stem and root	Transcriptional regulation of genes encoding microtubule- and actin-associated proteins	[81]
**Wind**	Fan-mediated/various exposure	*Solanum lycopersicum*	Stem	Restricted stem elongation	[82]
	Fan-mediated/6 h per day	*Arabidopsis thaliana*	Stem	Affected plant growth and phenology	[83]
	Fan-mediated/6–16 h per day		Stem	Impacted branching degree and fecundity	[59]
**Waves**	Flow flume system/20 s	Aquatic species	Stem/leaf/petioles	Negative correlation between avoidance and tolerance	[84]
**Flexure**	Various manual flexions/26 days	*Nicotiana tabacum*	Stem and leaves	Shorter, thicker stems with a lower Young’s modulus	[85]
	Daily manual flexure/90 s, for 72 days		Stem	Higher mass allocation to roots	[86]
	Stick-mediated strokes/daily, for 20 days	*Solanum lycopersicum*	Stem	Increase in root/shoot dry weight ratios	[87]
	Stick-mediated flexing/1 min, for 6 months	*Pinus sylvestris*	Stem and root	Reduced shoot height, higher root cross-sectional area and more lateral roots	[88]
**Vibrations**	Toothbrush/one minute per day, for 49 days	*Capsella bursa-pastoris*	Entire shoot	Increase in root/shoot biomass, accelerated senescence	[89]
**Rubbing**	Finger rubbing/once daily, for 5 days	*Phaseolus vulgaris*	Stem	Reduced first internodes length, thicker stems, reduced hollowing of the first internodes	[90]
	Finger rubbing/10 s	*Solanum lycopersicum*		Lignification-driven inhibited internode elongation	[91]
**Touch**	Water spray/Seconds	*Arabidopsis thaliana*		*TOUCH* genes-driven cell expansion	[92]
**Touch and brushing**	Hand touching, paint brushing/8–10 days			Reduced stem height, pivotal role of the RNA Polymerase-Associated Factor 1 Complex	[93]
**Brushing**	Paint brushing/10–20 s, for 7 days			Reduced inflorescence stem height, pivotal role for the pectic cell wall Arabinans	[94]
**Bending**	Tying around 90° mesh/5–6 months	*Populus nigra*	Woody taproot	Lateral root formation toward convex stretched side, lignification of concave compressed side (RW formation), root sector/side-specific hormonal profiles	[28,95]
**Bending**	Hook development model/N/A	*Arabidopsis thaliana*	Hypocotyl	Cellulose and PIN are essential for hook formation, auxin and pectin methylesterification crosstalk	[96,97]
**Gravity/bending**	Manual bending/Transient		Root	Lateral root initiation	[61,98]
**Barrier exposure**	Barrier, waving assay/N/A			Rapid and transient increases in cytosolic Ca^2+^, ROS production	[61]
**In vitro barrier**	Barrier exposure/6–30 h			Rapid obstacle avoidance forming a ‘step-like’ growth pattern	[63]
**Obstacle exposure**	Blades/200 min			PIN-mediated polar auxin transport facilitates root bending during obstacle avoidance	[99]
**Compacted soil**	Artificial macropores/4 months	*Triticum aestivum*	Root tip	Growth towards favorable soil conditions	[100]
	Agricultural machinery/7 days		Root	Invaginations and cortex cell deformation	[101]
	Dense containers/14 days	*Hordeum vulgare*	Root	Reduced total root length and leaf area, and altered biomass partitioning	[102]
	Drying/48 h	*Zea mays*	Root	Highly decreased root elongation and diameter	[103]
**Rigid pores**	Photoelastic disks/5 days	*Cicer arietinum*	Root	No significant growth reduction	[104]
**Rigid tubes**	Growth through narrow gap/24 h	*Zea mays*	Root apex	Atypical oblique divisions of the root cap cells	[105]

## Data Availability

Not applicable.
